# Not all migrants are the same: geographic origin and long-term outcomes after first-episode psychosis—a retrospective cohort study

**DOI:** 10.7717/peerj.21391

**Published:** 2026-07-24

**Authors:** Alba Toll, Sara Silles, Yolanda Manzano, Miriam Gregorio, Sandra Cepedello, Marta Niubó, Cristóbal Díez-Aja, Rebeca Hernández-Antón, Lara Moreno, Mariano Viotti, Leyda Cortés, Blanca Huedo, Jennifer García, Ariadna Moreno, Rosa Hernández-Ribas, Javier Labad

**Affiliations:** 1 Instituto de Salud Carlos III, Centro de Investigación Biomedica en Red de Salud Mental (CIBERSAM), Barcelona, Spain; 2 Department of Mental Health and Addictions, Consorci Sanitari del Maresme, Hospital Universitari de Mataró, Mataró, Spain

**Keywords:** Migration, First-episode psychosis, Treatment adherence, Disengagement, Antipsychotics, Health disparities, Mental health services

## Abstract

**Background:**

Migration is a well-established risk factor for psychosis, yet limited research has examined long-term clinical trajectories following first-episode psychosis (FEP) in migrant populations within Southern European settings. This study investigates how country of origin and time since migration influence psychiatric outcomes in a naturalistic FEP cohort.

**Methods:**

A 5-year longitudinal study was conducted on 184 patients with FEP attending the Early Intervention Service from Hospital Universitari de Mataró. Patients were grouped by geographic origin (Spanish, Latin American, Maghrebi, Sub-Saharan) and migration recency (≤5 years *vs*. >5 years). Primary outcomes included treatment adherence, use of long-acting injectable (LAI) antipsychotics, relapse, and service disengagement. Logistic regression and survival analyses were used to identify associations.

**Results:**

Maghrebi origin was associated with lower treatment adherence (OR = 0.32, 95% CI [0.12–0.89], *p* = 0.030). In time-to-event analyses, Maghrebi patients showed a higher hazard of service disengagement compared with Spanish-born patients (HR = 2.42, 95% CI [1.10–5.34], *p* = 0. 028). Recent migrants (≤5 years in Spain) also had a significantly increased hazard of disengagement (HR = 2.89, 95% CI [1.30–6.40], *p* = 0.009). Sub-Saharan origin was strongly associated with earlier initiation of long-acting injectable antipsychotics (HR = 3.40, 95% CI [1.58–7.32], *p* = 0.002), alongside higher overall odds of LAI use. No significant differences were observed in relapse rates across groups.

**Conclusions:**

Both migrant origin and recent arrival were associated with differences in psychiatric care trajectories following FEP. These findings highlight the need to consider adaptation of treatment approaches for migrant populations according to their origin and migration context in order to improve long-term outcomes.

## Introduction

Migration is a growing global phenomenon that increasingly intersects with mental health. Maresme region in Catalonia (Spain), like many areas in Southern Europe, has experienced a sharp rise in its foreign-born population. As of January 2023, 12.95% of Maresme residents were foreign nationals, with a demographic profile that is younger and more predominantly male than the local population (Consell Comarcal del Maresme, 2023). These demographic shifts call for a better understanding of how migration influences mental health trajectories, particularly in the early stages of severe mental illness.

There is robust evidence that migration is associated with an increased risk of developing psychotic disorders. Meta-analyses and multicentric cohort studies suggest that psychosocial stressors related to pre-migration adversity, traumatic migration processes, and post-migration challenges—including acculturative stress, discrimination, and marginalization—contribute to this elevated risk (Selten, van der Ven & Termorshuizen, 2020; Morgan, Knowles & Hutchinson, 2019). Reported odds ratios for psychosis in migrants and their descendants range from 1.6 to 1.9 across different populations and host countries (Jongsma et al., 2021; Tarricone et al., 2022). This risk is even more pronounced among visible minority groups and those exposed to childhood adversity, such as maltreatment or social exclusion (Varese et al., 2012; Selten & Cantor-Graae, 2005). The theory of “social defeat”—the chronic experience of being excluded from the majority group—has been proposed as a psychological and biological mechanism contributing to increased vulnerability to psychosis (Selten & Cantor-Graae, 2005).

In addition to heightened incidence, studies have shown that migrants are more likely to experience language barriers, delayed help-seeking, and reduced access to primary care pathways (Allegri, Belgiojoso & Rimoldi, 2025). Instead, they often enter psychiatric services through emergency departments or involuntary admissions, especially during first episodes psychosis (FEP) (Jongsma et al., 2021; Tarricone et al., 2022). These coercive and reactive pathways can disrupt the establishment of therapeutic alliance and contribute to long-term disengagement from mental health care (O’Brien, Fahmy & Singh, 2009; Kreyenbuhl, Nossel & Dixon, 2009).

Moreover, it has been suggested that potential differences in illness phenomenology could also influence care engagement. One Canadian study showed ethnic variations in clinical presentation among individuals with FEP, where Afro-Caribbean and Central/South American patients exhibited more severe negative symptoms compared to Euro-Canadian individuals at first presentation (Van der Ven et al., 2012). Additionally, pathways to care during FEP are shaped by culturally mediated illness attributions. It has been shown that Asian and Black patients were significantly more likely than White patients to seek help from faith-based organisations—although this did not delay psychiatric contact—highlighting the complexity of care-seeking behaviours across ethnic groups (Singh et al., 2015).

Ethnic disparities in care trajectories have also been widely reported. For example, Black African and Caribbean patients in the UK have a significantly increased likelihood of police involvement, compulsory hospitalization, and reduced contact with general practitioners at illness onset (Archie et al., 2010; Oduola et al., 2019), with systematic review evidence further supporting ethnic differences in pathways to care, particularly regarding police and general practitioner involvement (Anderson et al., 2014). These findings suggest that structural and systemic factors influence access to care beyond individual clinical characteristics.

Furthermore, real-world clinical data reveal ethnic inequities in treatment continuity. In a Canadian study of FEP patients (Nikolitch, Ryder & Jarvis, 2018), the authors found that Black patients had significantly lower rates of follow-up attendance compared to other groups, even when clinicians did not perceive differences in cooperation or insight. These findings underscore that disengagement may be more strongly linked to systemic barriers and patient mistrust than to individual attitudes or clinical presentation.

While much of the literature has focused on the incidence and pathways to care, less attention has been given to the long-term clinical course of psychosis among migrant patients, especially in Southern European or Mediterranean health systems. In particular, outcomes such as adherence to treatment, use of long-acting injectable (LAI) antipsychotics, relapse, and service disengagement remain underexplored. Moreover, few studies have simultaneously accounted for both country of origin and time since migration—two factors that may independently and interactively shape the illness trajectory.

This study aims to address this gap by analysing a naturalistic cohort of patients with FEP treated in the public mental health network of Maresme region in Catalonia (Spain). Specifically, we examine whether geographic origin and time since arrival in Spain are associated with differences in adherence, LAI initiation, relapse, and disengagement over a 5-year follow-up. We hypothesize that patients of migrant origin—particularly those recently arrived—will show lower adherence to treatment, higher rates of service disengagement, and may receive LAIs more frequently and earlier in the course of illness. By exploring these associations, we aim to contribute to a more nuanced understanding of the interplay between migration, ethnicity, and long-term psychiatric outcomes.

## Materials and Methods

### Study design and participants

The study included 184 patients with a diagnosis of first-episode psychosis (FEP) treated within the public mental health network of Maresme region in Catalonia (Spain). Patients were identified at cohort entry at the time of inclusion in the Early Intervention Service (EIS) for Psychosis of the Department of Mental Health at Hospital de Mataró. Consecutive incident cases were enrolled between June 1st, 2018 and March 31st, 2020, corresponding to the program inclusion period. All participants were subsequently followed for up to 5 years from the date of EIS entry, allowing the assessment of clinical outcomes over a 5-year follow-up window. No active recruitment was performed, as this was a retrospective study including all consecutive patients attended by the EIS during the inclusion period; therefore, no refusals were recorded.

Mental health care in Catalonia is provided through a universal, publicly funded healthcare system, ensuring free access at the point of delivery. Patients with FEP are managed within a specialized early psychosis care pathway, which follows the principles of Early Intervention in Psychosis, including rapid access to services, multidisciplinary follow-up, and continuity of care across inpatient and outpatient settings. Maresme region is characterized by a rapidly growing and socioeconomically diverse migrant population, providing a relevant setting to explore the interaction between migration and early psychosis.

FEP was defined as the presence of new-onset disorganized behaviour accompanied by hallucinations or delusions, not attributable to substance use or other medical condition, and meeting DSM-IV-TR criteria for a psychotic disorder (schizophrenia, bipolar disorder with psychotic features, major depressive disorder with psychotic features, schizophreniform disorder, brief psychotic disorder, delusional disorder, or psychosis not otherwise specified). Diagnoses were established using the Structured Clinical Interview for DSM-IV-TR Axis I Disorders (SCID-I).

Exclusion criteria were previous diagnosis of intellectual disability (IQ < 70) and/or active neurological or medical conditions that could explain the presenting symptoms.

The study was approved by the local Ethics Committee of Hospital de Mataró (Barcelona, Spain; protocol number 1/18). All participants, or their legal guardians when appropriate, received verbal and written information and provided written informed consent prior to enrolment.

### Clinical assessment and demographic data

The following sociodemographic variables were recorded: sex, age, country of origin, time since migration (in years), and educational level. The clinical assessment at baseline included initial diagnosis, which was established using the Structured Clinical Interview for DSM-IV-TR Axis I Disorders (SCID-I) and substance use (tobacco, alcohol, cannabis and cocaine) as dichotomic variables. At the 5-year follow-up, we recorded the number of patients who had initiated long-acting injectable (LAI) antipsychotics, including LAI type and time to initiation, as well as treatment adherence and relapse, both coded as dichotomous variables with corresponding time-to-event information.

Patients were categorized according to geographic origin into four groups: Spanish-born, Latin American, Maghrebi, and Sub-Saharan. Geographic origin was treated as a broad sociogeographic proxy variable rather than as a cultural or biological category. In this study, these groupings likely capture heterogeneous differences in structural position, including migration trajectories, social disadvantage, language barriers, and patterns of access to care. In addition, migrant patients were further classified based on their time since arrival in Spain, distinguishing between recent migrants (≤5 years since migration) and longer-settled migrants (>5 years). This threshold was selected *a priori* based on prior literature indicating that the early post-migration period is characterized by heightened psychosocial stress, cultural and linguistic barriers, and greater vulnerability related to settlement and health-care navigation. Previous research has used a 5-year cut-off to define recent migration and to capture this more unstable phase of post-migration adaptation (Moore, 2018).

Four main clinical outcomes were examined over the 5-year follow-up period: (1) treatment adherence; (2) initiation of long-acting injectable (LAI) antipsychotics; (3) relapse with psychiatric re-hospitalization; and (4) disengagement from mental health services.

Treatment adherence was assessed based on clinical records and medication continuity and was coded as a dichotomous variable using documented clinician evaluations throughout follow-up. This assessment integrated information on appointment attendance, reported medication adherence, and pharmacy records when available.

Disengagement from services was defined as loss of contact with mental health care for a sustained period exceeding routine appointment delays. Specifically, disengagement was operationalized as failure to attend three consecutive scheduled clinical appointments over a period of 6 months. Planned transfers to other services and documented geographic relocations were not classified as disengagement.

Relapse was defined as clinical deterioration requiring psychiatric hospital readmission, as documented in electronic clinical records.

Although reliance on routinely collected clinical records may introduce some degree of documentation-related misclassification, this approach reflects real-world clinical practice and was applied consistently across all study groups. In addition, within the Early Intervention Service, follow-up was conducted according to routine standardized care procedures, including scheduled multidisciplinary visits, systematic documentation in the electronic clinical record, and, for treatment adherence, verification of medication dispensing through pharmacy records.

### Statistical analysis

Data distribution normality was assessed with the Kolmogorov–Smirnov test. Descriptive statistics were used to summarize sociodemographic and clinical characteristics of the sample. Differences between groups were assessed using chi-square tests for categorical variables and ANOVA or Kruskal–Wallis tests for continuous variables, depending on normality.

Logistic regression models were conducted to examine the association between geographic origin and key clinical outcomes, including treatment adherence, initiation of long-acting injectable (LAI) antipsychotics, relapse requiring psychiatric re-hospitalization, and disengagement from mental health services.

All main models were adjusted *a priori* for relevant sociodemographic and clinical covariates, including age, sex, educational level, and substance use (tobacco, alcohol, cannabis, and cocaine). Use of LAI antipsychotics was additionally included as a covariate in models of relapse and service disengagement.

Diagnostic category was not included in the primary multivariate models to reduce the risk of model overfitting and instability, given the limited sample size within specific diagnostic subgroups. However, to assess the robustness of the main findings and address potential confounding by diagnosis, sensitivity analyses were conducted in which logistic regression models were additionally adjusted for diagnostic category. Diagnoses were grouped into schizophrenia-spectrum disorders, affective psychoses, and substance-induced psychosis to ensure adequate statistical power and model stability.

Survival analyses (Kaplan–Meier curves and log-rank tests) were used to examine time-to-event outcomes, including time to LAI initiation, time to relapse (defined as psychiatric re-hospitalization), and time to disengagement. In addition, Cox proportional hazards regression models were fitted to estimate adjusted hazard ratios while accounting for relevant sociodemographic and clinical covariates. The proportional hazards assumption was assessed for all Cox models and no relevant violations were detected. A complementary 1—survival function was used to visualize LAI initiation curves for better interpretability. Migrant patients were also stratified by time since arrival in Spain (≤5 years *vs*. >5 years) to assess the impact of recent migration on clinical outcomes.

Statistical significance was set at *p* < 0.05. All analyses were performed using SPSS version (IBM, Armonk, NY, USA) 25.0.

### Sample size considerations and events per variable

This was a retrospective cohort study including all consecutive patients with first-episode psychosis enrolled in the Early Intervention Service during the study inclusion period; therefore, no *a priori* sample size calculation was performed. The final sample size was determined by the total number of eligible patients meeting inclusion criteria.

To assess the adequacy of the multivariable models, we considered the events-per-variable (EPV) rule of thumb commonly recommended for logistic and survival regression analyses, which suggests a minimum of approximately 10 outcome events per estimated covariate to reduce overfitting and model instability.

Across outcomes, the number of observed events ranged from 40 (disengagement) to 80 (LAI initiation), with intermediate values for relapse and non-adherence. Approximate events-per-variable (EPV) values in the Cox models were 3.5 for disengagement, 5.4 for readmission, and 7.6 for LAI initiation. The EPV therefore varied across outcomes. Models examining LAI initiation approached commonly recommended EPV thresholds, whereas models for adherence and service disengagement involved fewer events per covariate, which may increase the risk of model instability and imprecise estimates. These limitations are reflected in the width of confidence intervals and were addressed through sensitivity analyses and cautious interpretation. Approximate events-per-variable (EPV) values in the Cox models were 3.5 for disengagement, 5.4 for readmission, and 7.6 for LAI initiation, reflecting variability in model stability across outcomes.

## Results

### Patient characteristics

A total of 184 patients with first-episode psychosis (FEP) were included in the study. The majority were male (*n* = 139; 75.5%), with a median age of 26.1 years (SD = 6.1). Regarding geographic origin, 64.7% were Spanish-born (*n* = 119), followed by Maghrebi (*n* = 29; 15.8%), Latin American (*n* = 14; 7.6%), Sub-Saharan (*n* = 13; 7.1%), and a smaller group from other regions (*n* = 9; 4.9%).

Diagnosis was distributed across the sample, with schizophrenia being the most common (28.8%), followed by psychosis not otherwise specified (20.1%), and bipolar disorder with psychotic features (16.3%). Substance use was frequent: 71.2% of patients had a history of use, particularly cannabis (60.3%) and tobacco (50.5%).

Significant differences by geographic origin were observed in sex distribution (*p* = 0.018), educational level (*p* = 0.001), and treatment adherence (*p* = 0.043). Specifically, the Maghrebi and Sub-Saharan groups had a markedly higher proportion of males (93.1% and 100%, respectively) compared to the Spanish-born group (70.6%). Educational attainment also varied notably: while 13.4% of Spanish-born patients had university-level education, no Sub-Saharan patients had completed upper secondary or university studies, and 92.3% had only primary education. Regarding treatment adherence, Maghrebi patients showed lower adherence rates (58.6%) compared to Spanish-born (81.5%) and Latin Americans (92.9%). Finally, significant differences were also found in the use of long-acting injectable (LAI) antipsychotics (*p* = 0.029), with the highest proportion of LAI users among Sub-Saharan patients (84.6%), compared to Maghrebi (48.3%) and Spanish-born patients (37.8%). Aripiprazole and paliperidone were the most commonly prescribed LAIs across all groups. Additional sociodemographic and clinical characteristics are detailed in Table 1.

**Table 1:** Clinical and sociodemographic characteristics by geographic origin.

	Total (*n* = 184)	Spanish-born (*n* = 119)	Latin American (*n* = 14)	Maghrebi (*n* = 29)	Sub Saharan (*n* = 13)	Others (*n* = 9)	*p*
**Age in years, m (sd)**	26.1 (6.1)	25.2 (5.9)	29.2 (5.5)	27.1 (5.8)	28.5 (7.6)	26.3 (5.9)	0.055
**Sex, *n* (% males)**	139 (75.5)	84 (70.6)	9 (64.3)	27 (93.1)	13 (100)	6 (66.7)	0.018
**Diagnosis, *n* (%)**							0.418
*Schizophreniform*	18 (9.8)	10 (8.4)	3 (21.4)	4 (13.8)	1 (7.7)	0 (0)	
*Schizophrenia*	53 (28.8)	32 (26.8)	4 (28.6)	10 (34.5)	4 (30.7)	3 (33.3)	
*Bipolar disorder*	30 (16.3)	19 (16)	3 (21.4)	4 (13.8)	2 (15.4)	2 (22.2)	
*Schizoaffective*	21 (11.4)	16 (13.4)	0 (0)	2 (6.9)	2 (15.4)	1 (11.1)	
*Substance induced psychosis*	15 (8.2)	9 (7.6)	2 (14.4)	3 (10.3)	0 (0)	1 (11.1)	
*Brief psychotic disorder*	0 (0)	0 (0)	0 (0)	0 (0)	0 (0)	0 (0)	
*Delusional disorder*	1 (0.5)	0 (0)	1 (7.1)	0 (0)	0 (0)	0 (0)	
*MDD with psychotic features*	9 (4.9)	8 (6.7)	0 (0)	0 (0)	1 (7.7)	0 (0)	
*Psychosis NOS*	37 (20.1)	25 (21.1)	1 (7.1)	6 (20.7)	3 (23.1)	2 (22.2)	
**Substance use, *n* (% users)**	131 (71.2)	83 (69.7)	11 (78.6)	24 (82.8)	7 (53.8)	6 (66.7)	0.357
*Tobacco*	93 (50.5)	61 (51.3)	5 (35.7)	16 (55.2)	5 (38.5)	6 (66.7)	0.525
*Alcohol*	58 (31.5)	35 (29.4)	7 (50)	10 (34.5)	3 (23.1)	3 (33.3)	0.554
*Cannabis*	111 (60.3)	70 (58.8)	7 (50)	22 (75.9)	7 (53.8)	5 (55.6)	0.411
*Cocaine*	27 (14.7)	16 (13.4)	3 (21.4)	6 (20.7)	2 (7.1)	0 (0)	0.551
**Education level, *n* (%)**							0.001
*Without regulated education*	3 (1.6)	0 (0)	0 (0)	3 (10.3)	0 (0)	0 (0)	
*Primary education*	77 (41.8)	42 (35.3)	3 (21.4)	17 (58.6)	12 (92.3)	3 (33.3)	
*Lower secondary education*	23 (12.5)	19 (16)	1 (7.1)	2 (6.9)	0 (0)	1 (11.1)	
*Vocational training*	38 (20.7)	24 (20.2)	4 (28.6)	5 (17.2)	1 (7.7)	4 (44.4)	
*Upper secondary education*	24 (13)	18 (15.1)	4 (28.6)	2 (6.9)	0 (0)	0 (0)	
*University*	19 (10.3)	16 (13.4)	2 (14.3)	0 (0)	0 (0)	1 (11.1)	
**LAI, *n* (% users)**	80 (43.5)	45 (37.8)	6 (42.9)	14 (48.3)	11 (84.6)	4 (44.4)	0.029
*Aripiprazole*	22 (12)	13 (10.9)	3 (21.4)	2 (6.9)	2 (15.4)	2 (22.2)	
*Paliperidone*	38 (20.7)	20 (16.8)	1 (7.1)	9 (31)	7 (53.8)	1 (11.1)	
*Risperidone*	13 (7.1)	8 (6.7)	2 (14.4)	1 (3.4)	2 (15.4)	0 (0)	
*Olanzapine*	5 (2.7)	3 (2.5)	0 (0)	2 (6.9)	0 (0)	0 (0)	
*Zuclopenthixol*	2 (1.1)	1 (0.8)	0 (0)	0 (0)	0 (0)	1 (11.1)	
**Treatment adherence, *n* (% users)**	145 (78.8)	97 (81.5)	13 (92.9)	17 (58.6)	10 (76.9)	8 (88.9)	0.043
**Relapse, *n* (%)**	65 (35.3)	41 (34.5)	3 (21.4)	11 (37.9)	7 (53.8)	3 (33.3)	0.513

**Note:**

Abbreviations: *n*, number of cases; sd, standard deviation; LAI, long-acting injectable antipsychotics; MDD, major depressive disorder; NOS, not otherwise specified.

Univariate odds ratios for each variable that would be included in the full adjusted logistic regression models are described in Table S1 from the Supplemental Material.

### Treatment adherence

In multivariate logistic regression analyses, Maghrebi patients had significantly lower odds of treatment adherence compared with Spanish-born patients (OR = 0.33; 95% CI [0.12–0.91]; *p* = 0.032). Tobacco use was also associated with lower adherence (OR = 0.27; 95% CI [0.10–0.72]; *p* = 0.009), whereas alcohol use showed a positive association with adherence (OR = 2.63; 95% CI [1.03–6.73]; *p* = 0.044) (Table 2).

**Table 2:** Multivariable logistic regression models for clinical outcomes.

Predictor	Adherence OR (95% CI)	*p*	LAI initiation OR (95% CI)	*p*	Relapse OR (95% CI)	*p*	Disengagement OR (95% CI)	*p*
Geographic origin								
Maghrebi	0.33 [0.12–0.91]	0.032	1.44 [0.59–3.50]	0.425	1.29 [0.49–3.44]	0.607	3.09 [1.11–8.60]	0.030
Sub-Saharan	0.71 [0.15–3.28]	0.662	10.36 [1.93–55.57]	0.006	1.23 [0.32–4.81]	0.764	1.61 [0.34–7.71]	0.551
Latin American	2.04 [0.23–17.93]	0.520	1.50 [0.46–4.92]	0.507	0.44 [0.10–2.00]	0.285	0.50 [0.06–4.41]	0.534
Age (per year)	1.02 [0.95–1.09]	0.578	0.97 [0.91–1.02]	0.219	0.95 [0.90–1.01]	0.125	0.98 [0.91–1.05]	0.551
Female sex	1.18 [0.40–3.49]	0.764	0.85 [0.38–1.92]	0.702	1.07 [0.44–2.57]	0.882	0.82 [0.28–2.44]	0.725
Education level	1.10 [0.80–1.51]	0.573	0.95 [0.74–1.21]	0.662	0.96 [0.74–1.26]	0.781	0.90 [0.66–1.24]	0.533
Cannabis use	0.97 [0.35–2.75]	0.960	1.09 [0.48–2.46]	0.835	0.45 [0.18–1.10]	0.081	1.04 [0.37–2.93]	0.941
Tobacco use	0.27 [0.10–0.72]	0.009	1.44 [0.66–3.12]	0.358	1.19 [0.50–2.81]	0.694	3.83 [1.43–10.30]	0.008
Alcohol use	2.63 [1.03–6.73]	0.044	1.21 [0.57–2.56]	0.615	1.91 [0.84–4.37]	0.124	0.38 [0.15–0.98]	0.045
Cocaine use	0.53 [0.18–1.55]	0.244	1.26 [0.50–3.16]	0.627	1.18 [0.43–3.22]	0.753	1.93 [0.65–5.69]	0.236
LAI use	–	–	–	–	3.34 [1.65–6.74]	0.001	0.72 [0.32–1.64]	0.433

**Note:**

Odds ratios (OR) with 95% confidence intervals (CI) are reported from fully adjusted logistic regression models. Overall model fit was modest, with Nagelkerke R^2^ values ranging from 0.12 to 0.20 across outcomes (adherence ≈ 0.19; LAI initiation ≈ 0.12; relapse ≈ 0.17; disengagement ≈ 0.20). All models were adjusted for age, sex, education level, and substance use (cannabis, tobacco, alcohol, and cocaine). Long-acting injectable (LAI) antipsychotic use was included as a covariate in the relapse and disengagement models, but not in the adherence model, and was the dependent variable in the LAI initiation model. Migration recency was examined in sensitivity analyses and is therefore not included in the main models. The reference category for geographic origin is Spanish-born.

### Use of LAI antipsychotics

**Figure 1:**
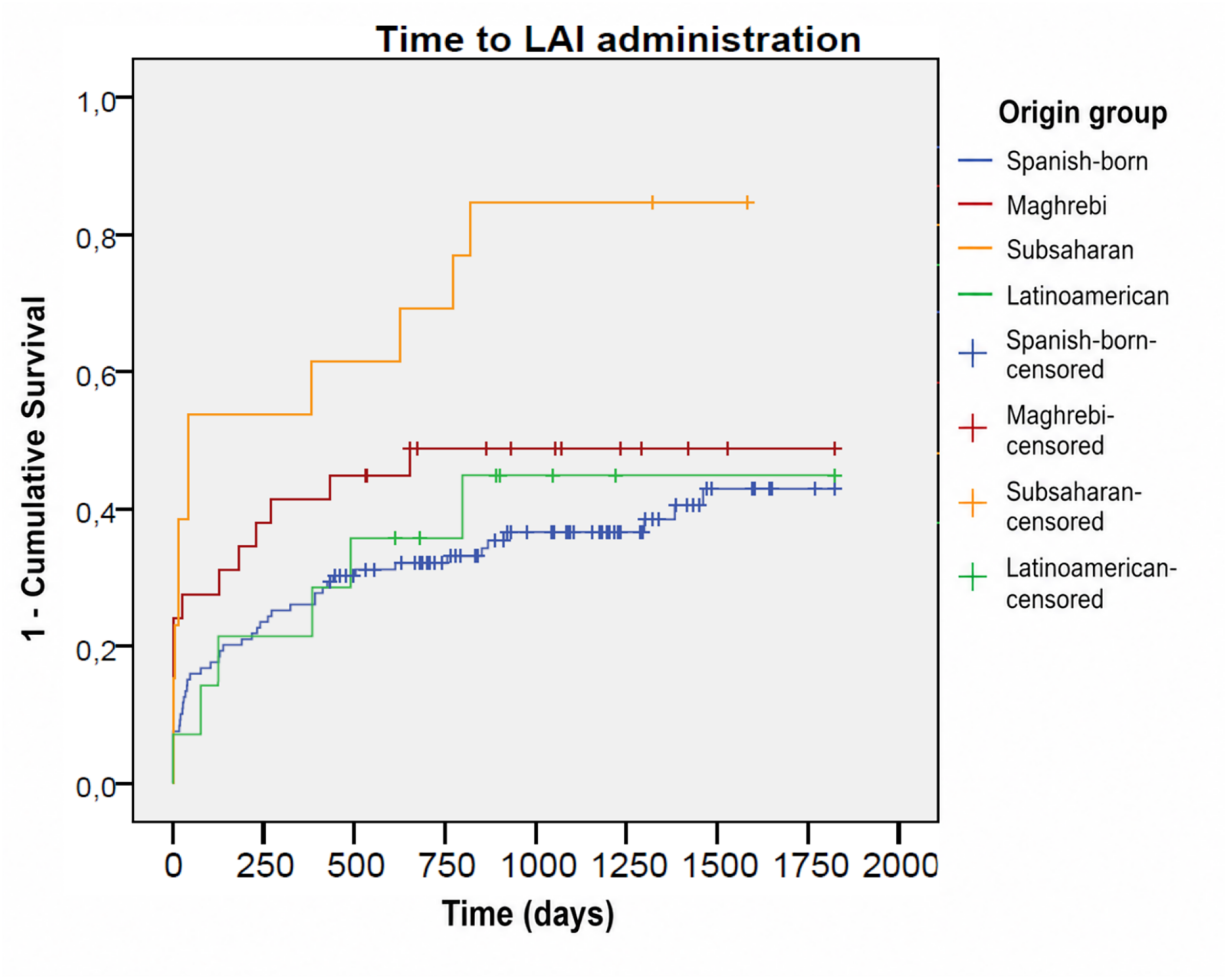
Time to initiation of long-acting injectable (LAI) antipsychotics according to geographic origin (Kaplan–Meier survival curves). Event was defined as initiation of a long-acting injectable antipsychotic. Patients who did not initiate LAI treatment were censored at the end of the 5-year follow-up or at their last recorded clinical contact. Survival curves were compared using the log-rank test. Abbreviations: LAI, long-acting injectable; Cum. survival, cumulative survival.

Sub-Saharan patients had significantly higher odds of initiating long-acting injectable (LAI) antipsychotic treatment during the 5-year follow-up compared with Spanish-born patients (OR = 10.36; 95% CI [1.93–55.57]; *p* = 0.006). This finding was consistent with the survival analysis; Kaplan–Meier analyses showed significant differences in time to LAI initiation across geographic origin groups (log-rank test *p* = 0.002), with earlier initiation observed in the Sub-Saharan group (Fig. 1). In adjusted Cox regression (Table S2), Sub-Saharan origin was associated with a higher hazard of LAI initiation compared with Spanish-born patients (HR = 3.40; 95% CI [1.58–7.32]; *p* = 0.002), after adjustment for age, sex, educational level, and substance use; no significant differences were observed for Maghrebi (HR = 1.33; 95% CI [0.68–2.57]; *p* = 0.405) or Latin American patients (HR = 1.39; 95% CI [0.57–3.35]; *p* = 0.467).

### Relapse and re-hospitalization

**Figure 2:**
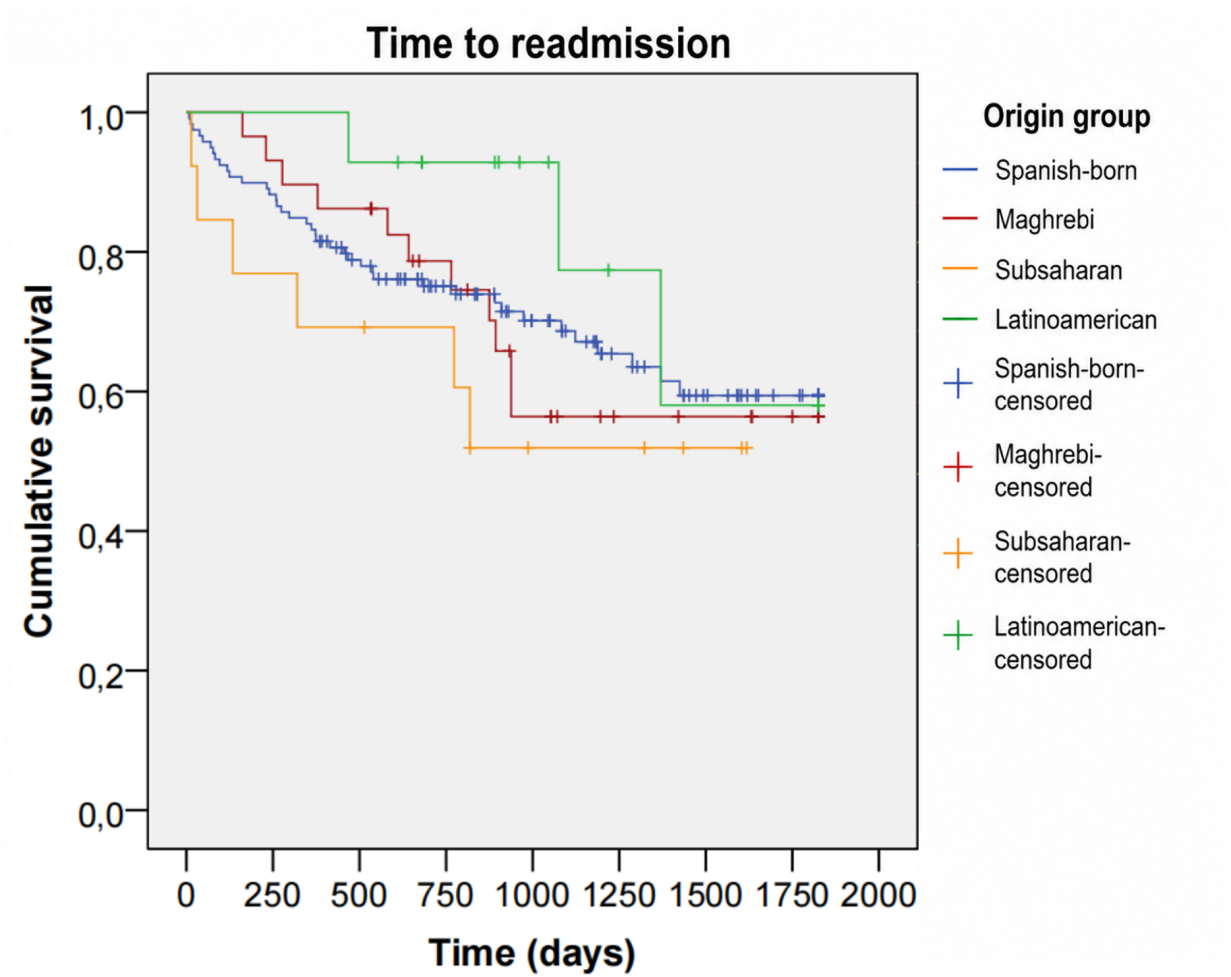
Time to relapse (psychiatric re-hospitalization) according to geographic origin (Kaplan–Meier survival curves). Event was defined as relapse requiring psychiatric re-hospitalization. Patients without relapse were censored at the end of the 5-year follow-up or at their last recorded clinical contact. Group differences were assessed using the log-rank test. Abbreviations: Cum. survival, cumulative survival.

No statistically significant differences in relapse were observed across geographic origin groups in either survival analyses (Fig. 2) or multivariate logistic regression models. This lack of association was confirmed in adjusted Cox proportional hazards models (Table S2), which did not identify geographic origin as an independent predictor of time to psychiatric re-hospitalization.

### Disengagement from EIS

**Figure 3:**
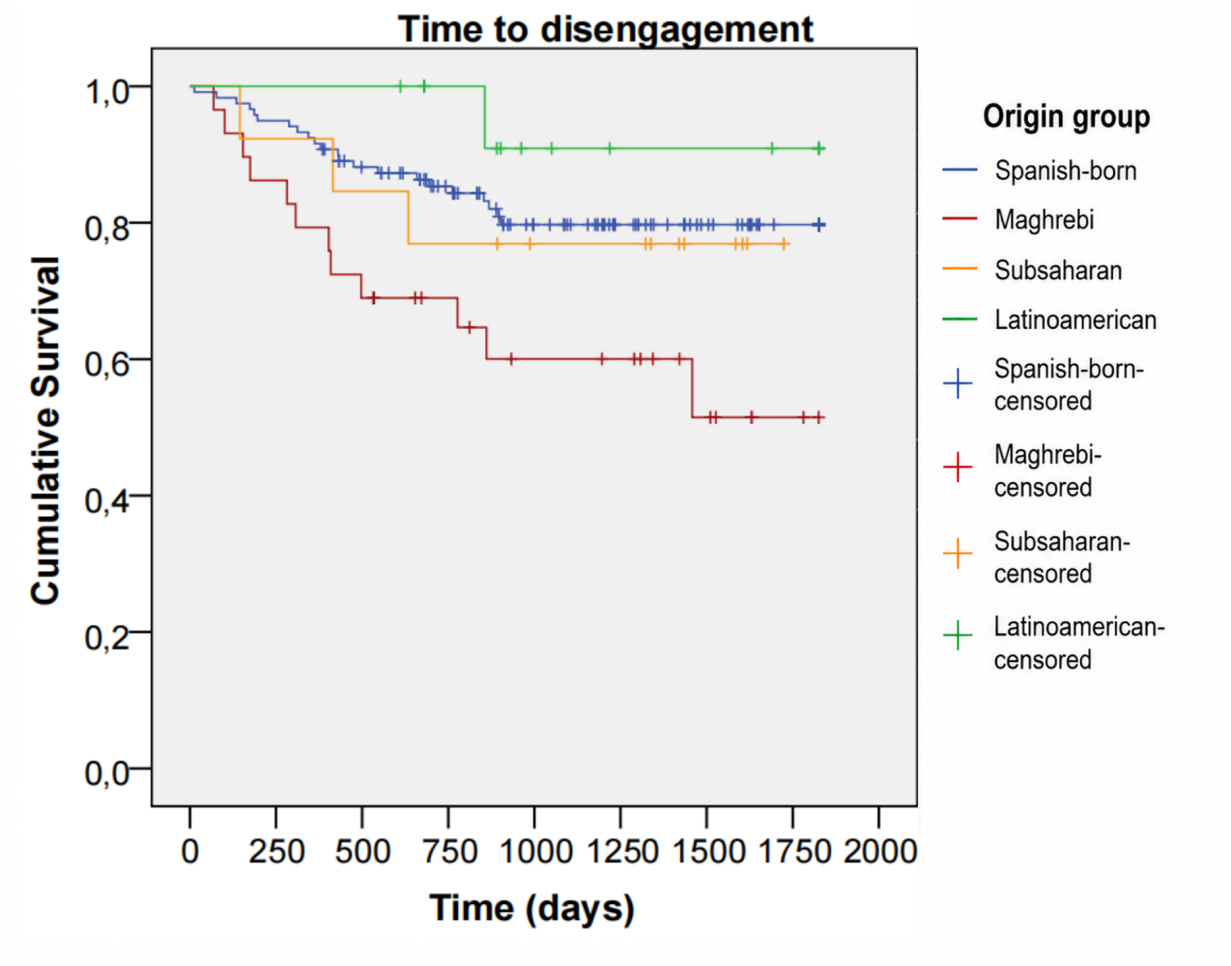
Time to service disengagement according to geographic origin (Kaplan–Meier survival curves). Event was defined as disengagement from mental health services, operationalized as loss of contact exceeding routine appointment delays. Planned transfers and documented relocations were censored. Survival curves were compared using the log-rank test. Abbreviations: Cum. survival, cumulative survival.

Maghrebi patients showed significantly higher odds of disengagement from EIS during the 5-year follow-up compared with Spanish-born patients (OR = 3.09; 95% CI [1.11–8.60]; *p* = 0.030). Tobacco use was also associated with increased odds of disengagement (OR = 3.83; 95% CI [1.43–10.30]; *p* = 0.008), whereas alcohol use was associated with lower odds of disengagement (OR = 0.38; 95% CI [0.15–0.98]; *p* = 0.045) (Table 2). Kaplan–Meier analysis demonstrated significant differences in time to disengagement across geographic origin groups (log-rank test *p* = 0.017), with earlier disengagement among Maghrebi patients (Fig. 3). In adjusted Cox proportional hazards models (Table S2), Maghrebi patients showed a significantly higher hazard of disengagement compared with Spanish-born patients (HR = 2.42; 95% CI [1.10–5.34]; *p* = 0.028), after adjustment for age, sex, educational level, substance use, and LAI treatment.

**Figure 4:**
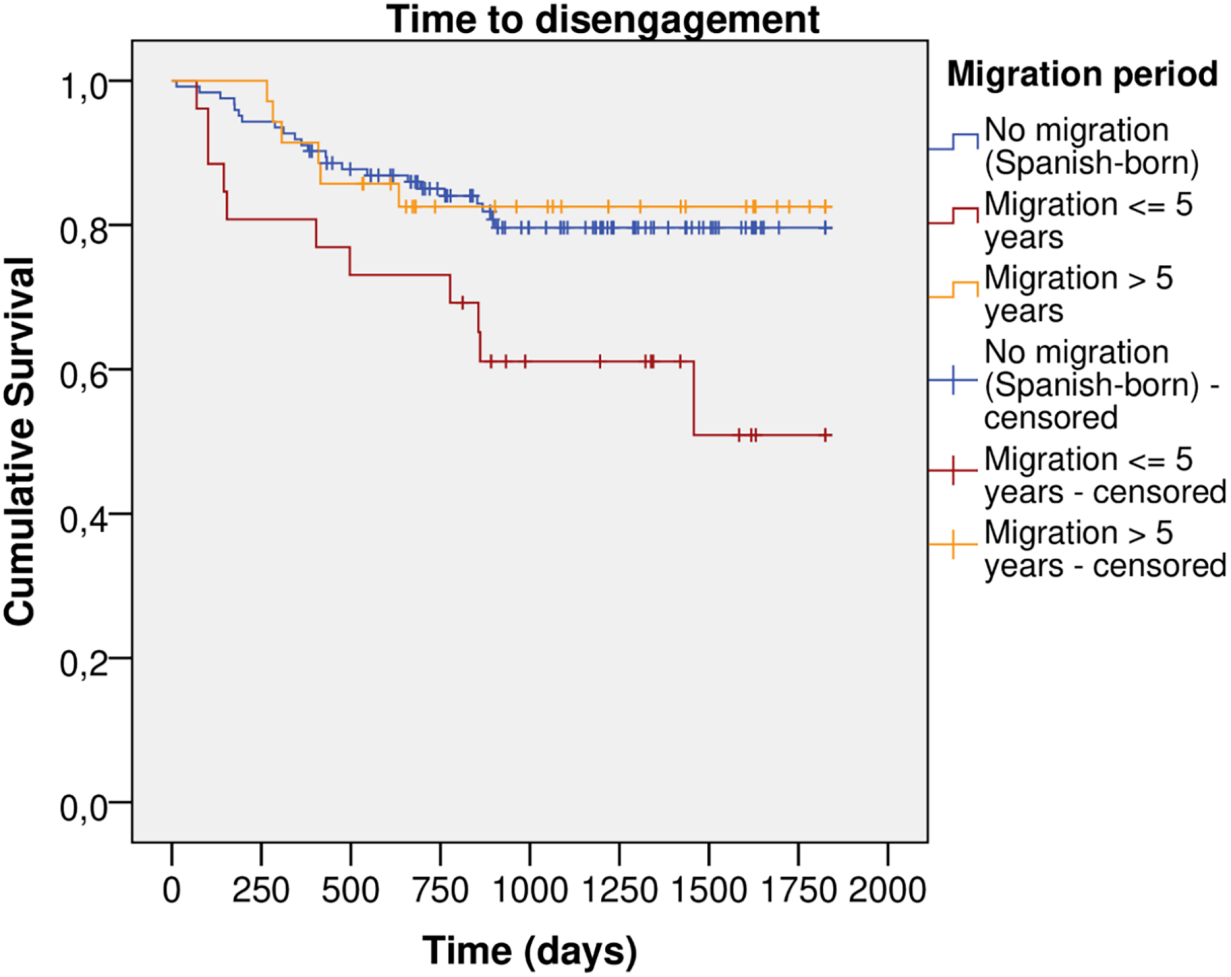
Time to service disengagement according to time since migration (≤5 years *vs*. >5 years) (Kaplan–Meier survival curves). Event was defined as disengagement from mental health services, operationalized as loss of contact exceeding routine appointment delays. Planned transfers and documented relocations were censored. Survival curves were compared using the log-rank test. Abbreviations: Cum. survival, cumulative survival.

Sensitivity analyses examining time since migration showed that individuals who had arrived in Spain within 5 years prior to first-episode psychosis onset had significantly higher odds of disengagement from EIS compared with those with longer residence duration (OR = 4.25; 95% CI [1.42–12.68]; *p* = 0.009), after adjustment for sociodemographic factors, substance use, and LAI treatment. Kaplan–Meier curves showed significant differences in time to disengagement across migration-timing groups (log-rank test *p* = 0.025), with earlier disengagement among individuals who had arrived in Spain within the 5 years prior to FEP onset (Fig. 4). In adjusted Cox regression models, recent migrants (≤5 years since arrival) showed a significantly higher hazard of disengagement compared with the non-migrant group (HR = 2.89; 95% CI [1.30–6.40]; *p* = 0.009), after adjustment for age, sex, educational level, substance use, and LAI treatment; no differences were observed for migrants with >5 years of residence (HR = 1.08; 95% CI [0.42–2.77]; *p* = 0.877).

### Sensitivity analyses adjusting for diagnostic category

Sensitivity analyses adjusting for diagnostic category yielded results comparable to the main models, with no material changes in the direction, magnitude, or statistical significance of the observed associations (Table S3).

## Discussion

This study sheds light on the long-term clinical trajectories of migrant patients following a first episode of psychosis (FEP) in a Mediterranean setting. Our findings reveal that both Maghrebi origin and recent migration (≤5 years) are significantly associated with poorer treatment adherence and higher risk of service disengagement over a 5-year period. In contrast, Sub-Saharan origin was strongly associated with earlier and higher odds of LAI use. These findings highlight the role of broader contextual factors in shaping psychiatric care trajectories among migrant populations.

The lower adherence observed in patients of Maghrebi origin is consistent with existing literature, which has highlighted challenges related to cultural stigma, divergent explanatory models of illness, and distrust toward public institutions (O’Brien, Fahmy & Singh, 2009; Kreyenbuhl, Nossel & Dixon, 2009). Moreover, these patients may face language barriers, limited health literacy, or misalignment between their expectations and those of clinicians. Tobacco use was also negatively associated with adherence, whereas alcohol use appeared to correlate positively. While this latter finding should be interpreted cautiously, it may reflect cultural patterns of substance use, where occasional alcohol consumption could be associated with greater acculturation and system familiarity, ultimately supporting care engagement.

Sub-Saharan origin was associated with a higher likelihood and earlier initiation of LAI treatment; however, these estimates should be interpreted cautiously given the small subgroup size, limited statistical power for subgroup analyses, and wide confidence intervals. Accordingly, interpretation should emphasize the direction and consistency of the observed effects rather than their precise magnitude. While it may indicate greater acceptability of LAIs, it may also reflect differential clinical decision-making processes or structural factors previously described in the literature, including clinicians’ assumptions about adherence and illness severity based on racial or ethnic background (Wang et al., 2024; Anderson et al., 2014; Oduola et al., 2019). This raises concerns about implicit bias in prescribing practices and highlights the importance of shared decision-making, especially in cross-cultural clinical contexts where communication asymmetries may undermine autonomy. However, these interpretations should be considered hypothesis-generating, as such mechanisms were not directly measured in the present study.

Although no significant differences were found in relapse rates (psychiatric re-hospitalization) across migrant groups, this finding should be interpreted within the broader context of disengagement. Patients lost to follow-up may have experienced relapse outside the formal healthcare system, leading to underreporting of this outcome. Some studies in this field have suggested that clinic records may underestimate early disengagement among Black patients, with only 24% attending more than half of scheduled appointments compared to 41% overall (Nikolitch, Ryder & Jarvis, 2018), highlighting the potential influence of non-clinical factors on service engagement.

Our study also confirms that recent migration (≤5 years) is a critical risk factor for disengagement. Migrants who had arrived in Spain shortly before psychosis onset had a 4-fold increased risk of disengaging from services. These findings align with the literature indicating that recency of migration interacts with multiple vulnerability factors—such as unstable housing, lack of social support, and bureaucratic obstacles—that compound difficulties in accessing and maintaining psychiatric care (Morgan, Knowles & Hutchinson, 2019; Kline & Thomas, 2018; Ouellet-Plamondon et al., 2015). Importantly, a sociocognitive framework suggests that disruptions in early developmental stages due to migration may lead to greater difficulty establishing trust and social integration, potentially impacting long-term psychiatric outcomes (Kirkbride et al., 2012).

Specifically for Latin American patients, our study showed no significant differences in adherence or disengagement compared to the Spanish-born population, although the small sample size may have limited statistical power. Prior studies in the United States found lower engagement with case management services among Latino patients with schizophrenia, especially among Spanish-speaking individuals with lower acculturation (Barrio et al., 2003). In that study, only 19% of Latinos with schizophrenia received case management compared to 30% of European-Americans, highlighting the potential role of language and cultural adaptation in service use. However, some protective factors, such as family support or the availability of bilingual services, may mitigate these disparities in certain contexts (Barrio et al., 2003).

In addition, one previous study (Kirkbride et al., 2012) emphasized the importance of age at immigration, showing that early migration during sensitive neurodevelopmental windows may increase psychosis risk, possibly *via* disruption of language development, peer relationships, and social stress processing. While our study did not directly assess age at migration, the proxy variable “time since migration” likely captures similar phenomena: individuals in the early stages of resettlement may not have had sufficient time to rebuild social networks or adapt to the host context, both of which are crucial for mental health stability.

Disparities in pathways to care remain a persistent concern across different health systems. Studies in the UK and North America have shown that ethnic minority patients are more likely to experience involuntary or emergency pathways into psychiatric care, often involving police or ambulance services (Oduola et al., 2019; Rotenberg et al., 2017). In addition, some evidence suggests that ethnic minorities may be less likely to receive timely mental health diagnoses or pharmacological treatment before their first psychotic episode (Coleman et al., 2019). Together, these findings highlight the influence of broader structural and systemic factors on access to and engagement with mental health services.

Altogether, our results advocate for an integrated transcultural approach to early psychosis care. This should involve training clinicians in cultural humility and structural competence, enhancing interpreter services, and building long-term trust through continuity of care. Interventions must be tailored to the specific needs of migrant populations, especially those recently arrived, recognizing how lived migration experiences—trauma, discrimination, adaptation—interface with clinical care trajectories.

### Strengths and Limitations

This study has several strengths. First, it provides long-term follow-up data from a naturalistic cohort of patients with first-episode psychosis (FEP) treated within a public mental health network in a demographically diverse region of Catalonia. The 5-year follow-up period allows for the examination of sustained outcomes such as treatment adherence, relapse, and disengagement—dimensions often underexplored in migrant populations. Second, the inclusion of both country of origin and time since migration as analytical variables allows for a more nuanced understanding of how migration-related factors interact with clinical trajectories. Few studies to date have incorporated both dimensions simultaneously. Finally, while our study is grounded in a specific regional context (Maresme area in Catalonia, Spain) and it may not be generalizable to other regions, the observed patterns are consistent with broader international trends and literature.

The study also presents some limitations. First, the retrospective design precludes control over data completeness and may introduce selection bias, as the cohort includes only individuals who accessed and remained in contact with the EIS. Patients who never reached mental health services or disengaged before formal program entry may therefore be underrepresented. Psychiatric diagnoses were established according to DSM-IV-TR criteria using the SCID-I, reflecting routine clinical practice at the time of recruitment. Although this approach ensures diagnostic reliability, it may limit direct comparability with more recent early psychosis studies employing DSM-5 or ICD-11 criteria. This study relied on retrospectively collected clinical data, which constrained the range of variables that could be included in the analyses. We prioritised the use of consistently documented, objective (“hard”) variables available across the full cohort, in order to minimise missing data and enhance comparability between groups. Consequently, potentially relevant clinical factors such as duration of untreated psychosis, baseline symptom severity, or insight were not included, as these were not systematically recorded for all patients. These variables may also act as explanatory mediators of the observed associations—particularly for disengagement and LAI initiation—rather than simple unmeasured confounders, and their absence limits causal interpretation. In addition, to preserve model stability given the number of outcome events, the inclusion of covariates in multivariate analyses was necessarily limited. These factors should be considered when interpreting the findings. A further limitation relates to sample size constraints in some migration subgroups, particularly the Latin American and Sub-Saharan groups. Although the overall cohort size was adequate for the main multivariable analyses, the number of events within certain subgroups was limited, which may reduce statistical power, affect model stability, and contribute to wide confidence intervals for some estimates (*e.g*., LAI initiation). These results should therefore be interpreted cautiously, with emphasis on effect direction and consistency rather than point estimates alone. To address this issue, we conducted sensitivity analyses including alternative model specifications, adjustment for diagnostic category, and analyses focusing on time since migration. The consistency of the main findings across these analyses supports the robustness of the observed associations. Finally, service disengagement was operationalized based on clinical records and may not fully capture patients who continued receiving care in other settings, moved outside the region, or experienced barriers to reengagement that were not documented.

### Conclusions

Our findings suggest that migrant origin and migration trajectory are associated with differences in clinical care trajectories following FEP. These results underscore the importance of adapting early psychosis interventions to the specific needs of migrant populations, taking into account their origin and migration context.

## Supplemental Information

10.7717/peerj.21391/supp-1 Supplemental Information 1 Univariable logistic regression analyses of factors associated with adherence, LAI initiation, relapse, and disengagement. Reference category for geographic origin is Spanish-born. Abbreviation: LAI, long-acting injectable

10.7717/peerj.21391/supp-2 Supplemental Information 2 Cox proportional hazards models for disengagement, readmission, and LAI initiation during 5-year follow-up. Abbreviation: LAI = long-acting injectable. Adjusted hazard ratios (HR) with 95% confidence intervals (CI) and *p* values for clinical outcomes. Reference categories: Spanish-born; male sex; no substance use; no LAI.

10.7717/peerj.21391/supp-3 Supplemental Information 3 Sensitivity analyses adjusting for diagnostic category. Abbreviation: LAI = long-acting injectable. Adjusted odds ratios (OR) with 95% confidence intervals (CI) and *p* values for clinical outcomes.

10.7717/peerj.21391/supp-4 Supplemental Information 4 Raw data.

## Additional Information and Declarations

### Data Availability

The following information was supplied regarding data availability:

The raw measurements are available in the Supplemental File.
