# Supplementary material for: Not all migrants are the same: geographic origin and long-term outcomes after first-episode psychosis—a retrospective cohort study
Source: PeerJ. 2026 Jul 24;14:e21391. doi: 10.7717/peerj.21391 (PMC13404132; doi:10.7717/peerj.21391)
Supplement: Supplemental Information 1 — Reference category for geographic origin is Spanish-born. Abbreviation: LAI, long-acting injectable [file peerj-14-21391-s001.docx]

| **Predictor** | **Adherence OR**  **(95% CI)** | | **p** | **LAI initiation OR**  **(95% CI)** | | **p** | **Relapse OR**  **(95% CI)** | **p** | **Disengagement OR**  **(95% CI)** | **p** |
| --- | --- | --- | --- | --- | --- | --- | --- | --- | --- | --- |
| Geographic origin |  | |  |  | |  |  |  |  |  |
| Maghrebi | | 0.32 (0.13–0.77) | 0.011 | | 1.54 (0.68–3.48) | 0.304 | 1.25 (0.54–2.91) | 0.599 | 3.11 (1.30–7.44) | 0.011 |
| Sub-Saharan | | 0.76 (0.19–2.98) | 0.689 | | 9.04 (1.92–42.68) | 0.005 | 1.76 (0.55–5.58) | 0.339 | 1.32 (0.34–5.21) | 0.689 |
| Latin American | | 2.95 (0.37–23.74) | 0.310 | | 1.23 (0.40–3.79) | 0.714 | 0.56 (0.15–2.12) | 0.393 | 0.34 (0.04–2.73) | 0.310 |
| Age (per year) | 1.01 (0.95–1.07) | | 0.718 | 1.00 (0.95–1.05) | | 0.912 | 0.95 (0.91–1.01) | 0.078 | 0.99 (0.93–1.05) | 0.675 |
| Female sex | 2.02 (0.79–5.20) | | 0.143 | 0.50 (0.25–1.02) | | 0.057 | 0.88 (0.43–1.82) | 0.738 | 0.48 (0.19–1.22) | 0.122 |
| Education level | 1.31 (1.00–1.73) | | 0.050 | 0.81 (0.66–1.00) | | 0.048 | 0.89 (0.71–1.10) | 0.279 | 0.75 (0.57–0.98) | 0.036 |
| Cannabis use | 0.45 (0.20–0.99) | | 0.047 | 1.56 (0.85–2.85) | | 0.151 | 0.83 (0.45–1.55) | 0.565 | 2.33 (1.06–5.13) | 0.035 |
| Tobacco use | 0.32 (0.15–0.69) | | 0.004 | 1.64 (0.91–2.95) | | 0.099 | 1.12 (0.61–2.07) | 0.714 | 3.30 (1.53–7.10) | 0.002 |
| Alcohol use | 1.22 (0.56–2.66) | | 0.616 | 1.47 (0.79–2.75) | | 0.227 | 1.52 (0.80–2.91) | 0.205 | 0.78 (0.36–1.70) | 0.537 |
| Cocaine use | 0.47 (0.19–1.16) | | 0.100 | 1.49 (0.66–3.37) | | 0.344 | 1.22 (0.52–2.86) | 0.643 | 2.03 (0.83–4.96) | 0.119 |
| LAI use | — | | — | — | | — | 3.19 (1.68-6.03) | <0.001 | 0.95 (0.47–1.93) | 0.888 |

**Table S1. Univariable logistic regression analyses of factors associated with adherence, LAI initiation, relapse, and disengagement**

Reference category for geographic origin is Spanish-born.

Abbreviation: LAI, long-acting injectable
