# Supplementary material for: Not all migrants are the same: geographic origin and long-term outcomes after first-episode psychosis—a retrospective cohort study
Source: PeerJ. 2026 Jul 24;14:e21391. doi: 10.7717/peerj.21391 (PMC13404132; doi:10.7717/peerj.21391)
Supplement: Supplemental Information 2 — Abbreviation: LAI = long-acting injectable. Adjusted hazard ratios (HR) with 95% confidence intervals (CI) and p values for clinical outcomes. Reference categories: Spanish-born; male sex; no substance use; no LAI. [file peerj-14-21391-s002.docx]

**Supplementary Table S2. Cox proportional hazards models for disengagement, readmission, and LAI initiation during 5-year follow-up.**

|  | **Disengagement** | | **Readmission** | | **LAI initiation** | | |
| --- | --- | --- | --- | --- | --- | --- | --- |
| **Variable** | **HR (95% CI)** | **p-value** | **HR (95% CI)** | **p-value** | **HR (95% CI)** | | **p-value** |
| Geographic origin |  |  |  |  |  | |  |
| Maghrebi | 2.42 (1.10–5.34) | 0.028 | 1.03 (0.50–2.13) | 0.939 | | 1.33 (0.68–2.57) | 0.405 |
| Sub-Saharan | 1.55 (0.40–5.93) | 0.524 | 1.38 (0.50–3.81) | 0.529 | | 3.40 (1.58–7.32) | 0.002 |
| Latin American | 0.53 (0.07–4.10) | 0.545 | 0.44 (0.12–1.54) | 0.198 | | 1.39 (0.57–3.35) | 0.467 |
| Age | 0.98 (0.92–1.05) | 0.544 | 0.95 (0.90–1.00) | 0.042 | | 0.98 (0.94–1.02) | 0.405 |
| Female sex | 0.93 (0.35–2.47) | 0.887 | 1.16 (0.57–2.35) | 0.687 | | 0.91 (0.48–1.75) | 0.783 |
| Education level | 0.90 (0.68–1.20) | 0.486 | 0.94 (0.75–1.17) | 0.561 | | 0.93 (0.77–1.12) | 0.459 |
| Cannabis use | 1.23 (0.50–3.05) | 0.654 | 0.64 (0.32–1.30) | 0.219 | | 1.16 (0.63–2.17) | 0.632 |
| Tobacco use | 2.67 (1.17–6.13) | 0.020 | 0.99 (0.50–1.95) | 0.980 | | 1.34 (0.75–2.37) | 0.321 |
| Alcohol use | 0.53 (0.25–1.15) | 0.108 | 1.66 (0.87–3.15) | 0.123 | | 0.98 (0.58–1.67) | 0.941 |
| Cocaine use | 1.52 (0.68–3.42) | 0.311 | 1.50 (0.70–3.22) | 0.302 | | 1.26 (0.68–2.36) | 0.463 |
| LAI initiation | 0.73 (0.37–1.45) | 0.371 | 2.31 (1.32–4.03) | 0.003 | | — | — |

Abbreviation: LAI= long-acting injectable.

Adjusted hazard ratios (HR) with 95% confidence intervals (CI) and p values for clinical outcomes.

Reference categories: Spanish-born; male sex; no substance use; no LAI.
