# Supplementary material for: Not all migrants are the same: geographic origin and long-term outcomes after first-episode psychosis—a retrospective cohort study
Source: PeerJ. 2026 Jul 24;14:e21391. doi: 10.7717/peerj.21391 (PMC13404132; doi:10.7717/peerj.21391)
Supplement: Supplemental Information 3 — Abbreviation: LAI = long-acting injectable. Adjusted odds ratios (OR) with 95% confidence intervals (CI) and p values for clinical outcomes. [file peerj-14-21391-s003.docx]

| **Variable** | **Adherence OR**  **(95% CI)** | **p** | **LAI initiation OR**  **(95% CI)** | **p** | **Relapse OR**  **(95% CI)** | **p** | **Disengagement OR**  **(95% CI)** | **p** |
| --- | --- | --- | --- | --- | --- | --- | --- | --- |
| Geographic origin |  |  |  |  |  |  |  |  |
| Maghrebi | 0.33 (0.12–0.92) | 0.034 | 1.37 (0.56–3.37) | 0.490 | 1.32 (0.49–3.59) | 0.586 | 3.06 (1.10–8.52) | 0.032 |
| Sub-Saharan | 0.73 (0.16–3.42) | 0.688 | 9.88 (1.81–54.05) | 0.008 | 1.04 (0.26–4.19) | 0.956 | 1.59 (0.33–7.74) | 0.568 |
| Latin American | 2.02 (0.23–17.80) | 0.527 | 1.56 (0.47–5.18) | 0.469 | 0.45 (0.10–2.04) | 0.301 | 0.52 (0.06–4.58) | 0.554 |
| Diagnosis |  |  |  |  |  |  |  |  |
| Affective psychosis | 1.11 (0.40–3.04) | 0.844 | 0.61 (0.27–1.37) | 0.230 | 2.40 (1.03–5.58) | 0.043 | 0.85 (0.30–2.37) | 0.751 |
| Substance-induced psychosis | 1.19 (0.27–5.32) | 0.823 | 0.64 (0.19–2.18) | 0.472 | 0.41 (0.07–2.30) | 0.310 | 0.79 (0.17–3.63) | 0.767 |
| Age (per year) | 1.02 (0.95–1.10) | 0.611 | 0.97 (0.91–1.03) | 0.257 | 0.96 (0.90–1.02) | 0.228 | 0.98 (0.91–1.05) | 0.586 |
| Female sex | 1.19 (0.40–3.54) | 0.75 | 0.80 (0.35–1.83) | 0.601 | 1.12 (0.46–2.74) | 0.805 | 0.81 (0.27–2.41) | 0.702 |
| Education level | 1.10 (0.80–1.52) | 0.567 | 0.94 (0.73–1.21) | 0.637 | 0.91 (0.69–1.21) | 0.523 | 0.90 (0.65–1.25) | 0.527 |
| Cannabis use | 0.98 (0.34–2.80) | 0.963 | 1.10 (0.48–2.52) | 0.823 | 0.49 (0.20–1.22) | 0.126 | 1.04 (0.36–2.97) | 0.948 |
| Tobacco use | 0.27 (0.10–0.72) | 0.009 | 1.46 (0.67–3.21) | 0.346 | 1.11 (0.46–2.65) | 0.816 | 3.89 (1.43–10.59) | 0.008 |
| Alcohol use | 2.61 (1.02–6.69) | 0.045 | 1.20 (0.57–2.53) | 0.638 | 1.97 (0.85–4.58) | 0.115 | 0.39 (0.15–0.99) | 0.047 |
| Cocaine use | 0.54 (0.18–1.58) | 0.259 | 1.19 (0.47–3.02) | 0.716 | 1.30 (0.47–3.61) | 0.616 | 1.88 (0.63–5.61) | 0.256 |
| LAI initiation | — | — | — | — | 3.70 (1.78–7.67) | <0.001 | 0.70 (0.30–1.62) | 0.402 |

**Supplementary Table S3. Sensitivity analyses adjusting for diagnostic category**

Abbreviation: LAI= long-acting injectable.

Adjusted odds ratios (OR) with 95% confidence intervals (CI) and p values for clinical outcomes.

Reference categories: Spanish-born; schizophrenia-spectrum disorders; male sex; no substance use; no LAI.
